# An Engineered Yeast Efficiently Secreting Penicillin

**DOI:** 10.1371/journal.pone.0008317

**Published:** 2009-12-15

**Authors:** Loknath Gidijala, Jan A. K. W. Kiel, Rutger D. Douma, Reza M. Seifar, Walter M. van Gulik, Roel A. L. Bovenberg, Marten Veenhuis, Ida J. van der Klei

**Affiliations:** 1 Molecular Cell Biology, Groningen Biomolecular Sciences and Biotechnology Institute (GBB), University of Groningen, Haren, The Netherlands; 2 Kluyver Centre for Genomics of Industrial Fermentation, Delft, The Netherlands; 3 Department of Biotechnology, Faculty of Applied Sciences, Delft University of Technology, Delft, The Netherlands; 4 DSM Biotechnology Centre, Delft, The Netherlands; 5 Synthetic Biology and Cell Engineering, Groningen Biomolecular Sciences and Biotechnology Institute (GBB), University of Groningen, Haren, The Netherlands; Instituto Butantan, Brazil

## Abstract

This study aimed at developing an alternative host for the production of penicillin (PEN). As yet, the industrial production of this β-lactam antibiotic is confined to the filamentous fungus *Penicillium chrysogenum*. As such, the yeast *Hansenula polymorpha*, a recognized producer of pharmaceuticals, represents an attractive alternative. Introduction of the *P. chrysogenum* gene encoding the non-ribosomal peptide synthetase (NRPS) δ-(L-α-aminoadipyl)-L-cysteinyl-D-valine synthetase (ACVS) in *H. polymorpha*, resulted in the production of active ACVS enzyme, when co-expressed with the *Bacillus subtilis sfp* gene encoding a phosphopantetheinyl transferase that activated ACVS. This represents the first example of the functional expression of a non-ribosomal peptide synthetase in yeast. Co-expression with the *P. chrysogenum* genes encoding the cytosolic enzyme isopenicillin N synthase as well as the two peroxisomal enzymes isopenicillin N acyl transferase (IAT) and phenylacetyl CoA ligase (PCL) resulted in production of biologically active PEN, which was efficiently secreted. The amount of secreted PEN was similar to that produced by the original *P. chrysogenum* NRRL1951 strain (approx. 1 mg/L). PEN production was decreased over two-fold in a yeast strain lacking peroxisomes, indicating that the peroxisomal localization of IAT and PCL is important for efficient PEN production. The breakthroughs of this work enable exploration of new yeast-based cell factories for the production of (novel) β-lactam antibiotics as well as other natural and semi-synthetic peptides (e.g. immunosuppressive and cytostatic agents), whose production involves NRPS's.

## Introduction

β-Lactam antibiotics (penicillins and cephalosporins) represent a class of important drugs of major clinical value. Their significant economical value is evident from the fact that β-lactam antibiotics contribute to over 40% of the total antibiotic market [1]. The industrial production of penicillin (PEN) occurs via fermentation using the filamentous fungus *Penicillium chrysogenum*. There is considerable interest in developing novel cell factories for the production of (new) β-lactam antibiotics, because of the intrinsic drawbacks of filamentous fungi for large scale industrial fermentations. Unicellular yeast species are very attractive alternatives as they have superior fermentation characteristics over filamentous fungi. Production of PEN and other β-lactam antibiotics in yeast will also provide new opportunities for highly sustainable production processes and the development of generic strategies to produce modified β-lactams and eventually other peptide antibiotics using the power of yeast genetics.

The yeast *Hansenula polymorpha*, a recognized producer of pharmaceuticals [2], represents an attractive alternative for PEN production. Advantages of this organism include the availability of very strong and regulatable promoters and excellent fermentation properties. An example includes the large scale industrial production of hepatitis B antigen [2]. Also, in this yeast peroxisomes can be massively induced. This is a favorable property to facilitate PEN production, which is known to involve peroxisomal enzymes.

**Figure 1:**
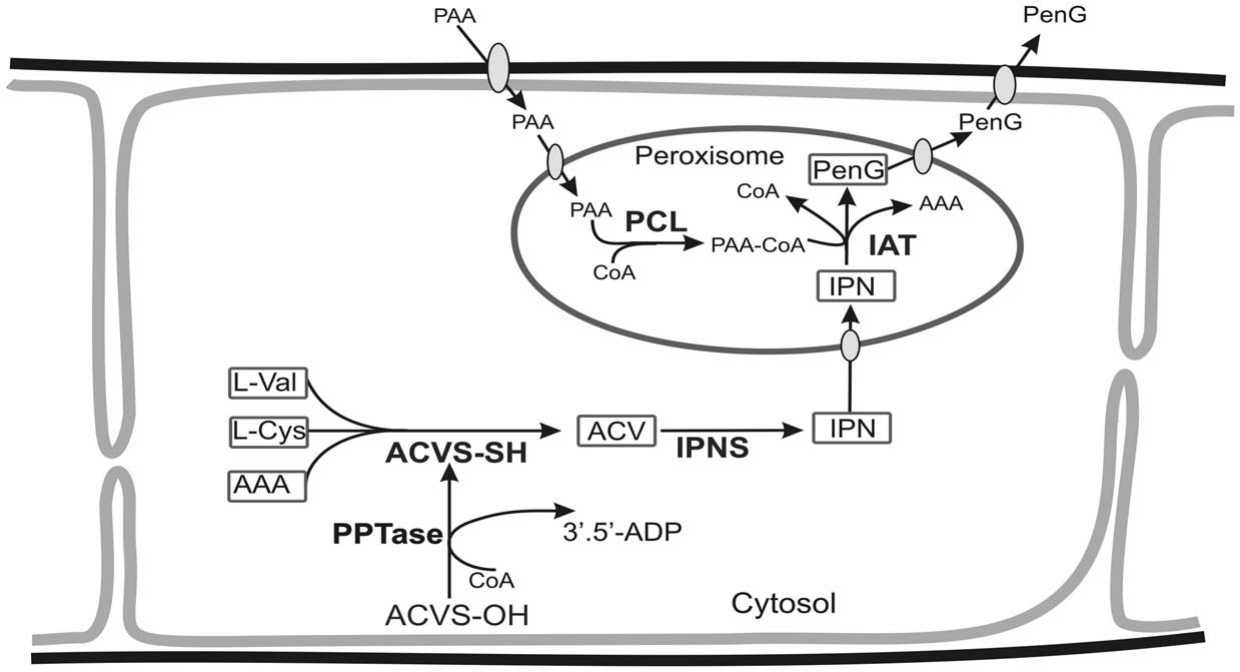
Schematic representation of the penicillin G (PenG) biosynthetic pathway in *P. chrysogenum*. In the cytosol, the enzyme ACVS (ACVS-OH) is activated by a PPTase into ACVS-SH. The active enzyme produces ACV from the three precursor molecules AAA, L-cysteine and L-valine. IPNS subsequently converts ACV into the β-lactam IPN, which is transported into peroxisomes. In this organelle PAA is activated by PCL into phenylacetyl CoA (PA-CoA), which is used by IAT to synthesize PenG from IPN. PenG is exported from the organelle and ultimately secreted into the medium. The precursors and intermediates of PenG biosynthesis pathway are boxed.

Adapting yeast to produce PEN requires the introduction of the complete PEN biosynthetic pathway in the organism. This involves the non-ribosomal peptide synthetase (NRPS) δ-(L-α-aminoadipyl)-L-cysteinyl-D-valine synthetase (ACVS), isopenicillin N synthase (IPNS), isopenicillin N acyl transferase (IAT) and phenylacetyl CoA ligase (PCL). Of these, ACVS and IPNS are cytosolic, whereas IAT and PCL are localized to peroxisomes (Fig. 1) [3]. Notably, ACVS belongs to a class of enzymes (NRPS's) that exclusively occurs in certain filamentous fungi and bacteria (*Actinomycetes, Bacilli*).

Here we show that we successfully engineered *H. polymorpha* to produce biologically active PEN. Our work involves major breakthroughs by showing:

1. the functional expression of an NRPS in yeast,
2. the successful reconstitution of the complete, compartmentalized PEN biosynthetic pathway in *H. polymorpha*
3. that PEN is highly efficiently secreted by *H. polymorpha*.
4. that the peroxisomal localization of the last two enzymes of the PEN biosynthesis pathway is important for efficient PEN production.

These achievements are of major significance for the development of novel yeast-based production platforms for the production of novel β-lactams and other important peptide-based pharmaceuticals.

## Results

### Functional Production of ACVS in *H. polymorpha*

**Figure 2:**
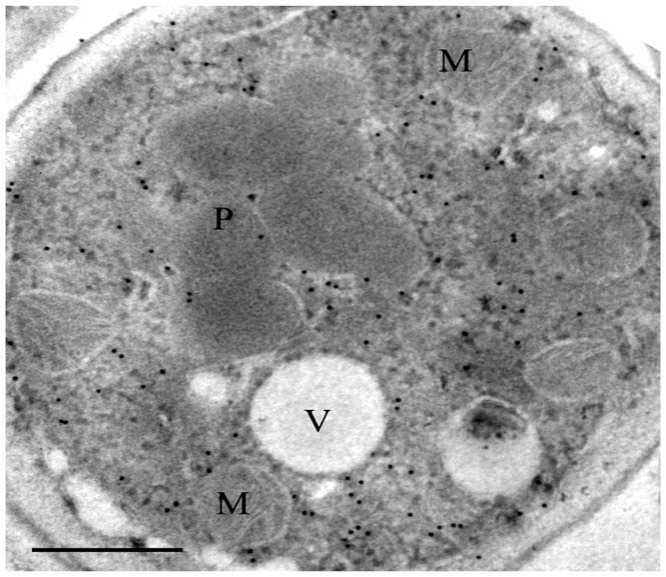
Subcellular localization of ACVS in *H. polymorpha*. Immunocytochemistry using anti-ACVS antibodies showing the presence of ACVS protein in the cytosol of strain HpPen4. Cells were fixed in 3% glutaraldehyde for 1 h on ice, dehydrated in an ethanol series and embedded in Lowicryl, polymerized by UV light. Post-staining was with 0.5% uranylacetate. M – mitochondrion; P – peroxisome; V – vacuole. The bar represents 0.5 µm.

**Figure 3:**
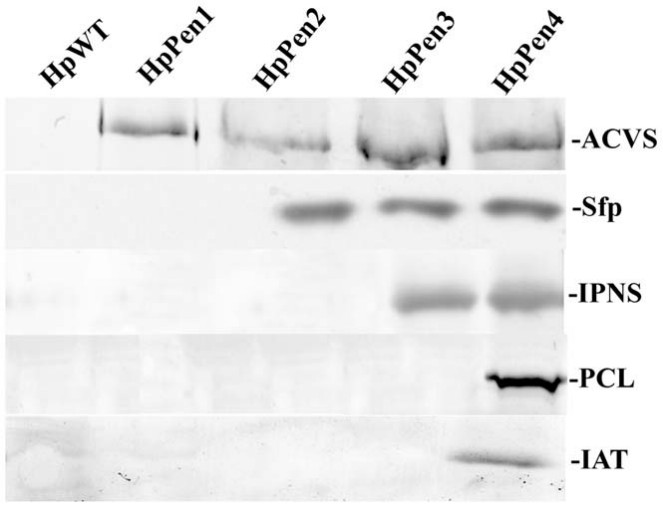
Expression of genes involved in penicillin G production in *H. polymorpha*. Western blot analysis demonstrating the presence of ACVS, Sfp, IPNS, IAT and PCL in cells of the various indicated *H. polymorpha* strains grown in batch cultures on methanol. The blots were decorated with the indicated antibodies except for Sfp, which was produced as a His_6_ tagged protein and detected by anti-His_6_ antibodies. Per lane 20 µg of protein was loaded, except for IPNS for which 2 µg of protein was used.

**Figure 4:**
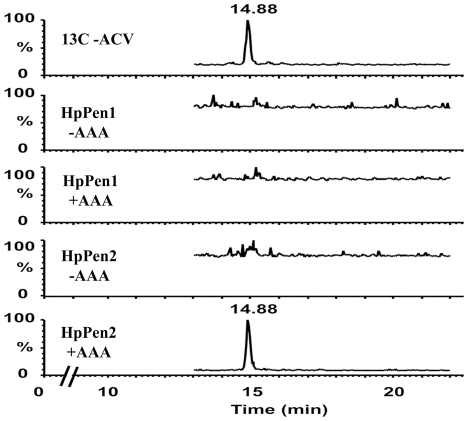
ACV production in HpPen2. Multiple reaction monitoring (MRM) chromatograms from ACV in extracts of strains HpPen1 and HpPen2 cells grown in batch cultures on methanol in the presence (+AAA) or absence (-AAA) of 8 mM AAA. The data show that ACV is only present in HpPen2 cells grown in the presence of AAA (lowest panel). An MRM chromatogram from the co-eluting ^13^C labeled ACV internal standard is included as control (upper panel).

In our approach to reprogram *H. polymorpha* to produce PEN, we first set out to generate a strain synthesizing enzymatically active ACVS, a 450 kD NRPS. Upon introduction of the *P. chrysogenum pcbAB* gene encoding ACVS in *H. polymorpha* (strain HpPen1), the protein was properly produced and localized to the cytosol (Fig. 2 and 3). However, when these cells were grown in the presence of the ACVS substrate α-aminoadipic acid (AAA), ACV production could not be demonstrated (Fig. 4). This was most likely related to the fact that activation of NRPS enzymes requires covalent attachment of a phosphopantetheinyl moiety to the peptidyl carrier protein domain of the enzyme [4], a reaction that is catalyzed by phosphopantetheinyl transferases (PPTases). Apparently, *H. polymorpha* does not contain a PPTase that is able to activate ACVS *in vivo*. Therefore, we introduced the *Bacillus subtilis* PPTase Sfp, which exhibits a broad substrate specificity [5], in HpPen1, thus generating strain HpPen2 (Fig. 3).

When HpPen2 was grown in the presence of AAA, ACV was indeed produced (Fig. 4), demonstrating that *B. subtilis* Sfp had activated ACVS. ACV was not observed when HpPen2 cells were grown in the absence of AAA (Fig. 4), which indicates that AAA is a limiting substrate in *H. polymorpha*, but can be taken up by the yeast cells from the cultivation medium.

### Secretion of a Bioactive β-Lactam by *H. polymorpha*

**Figure 5:**
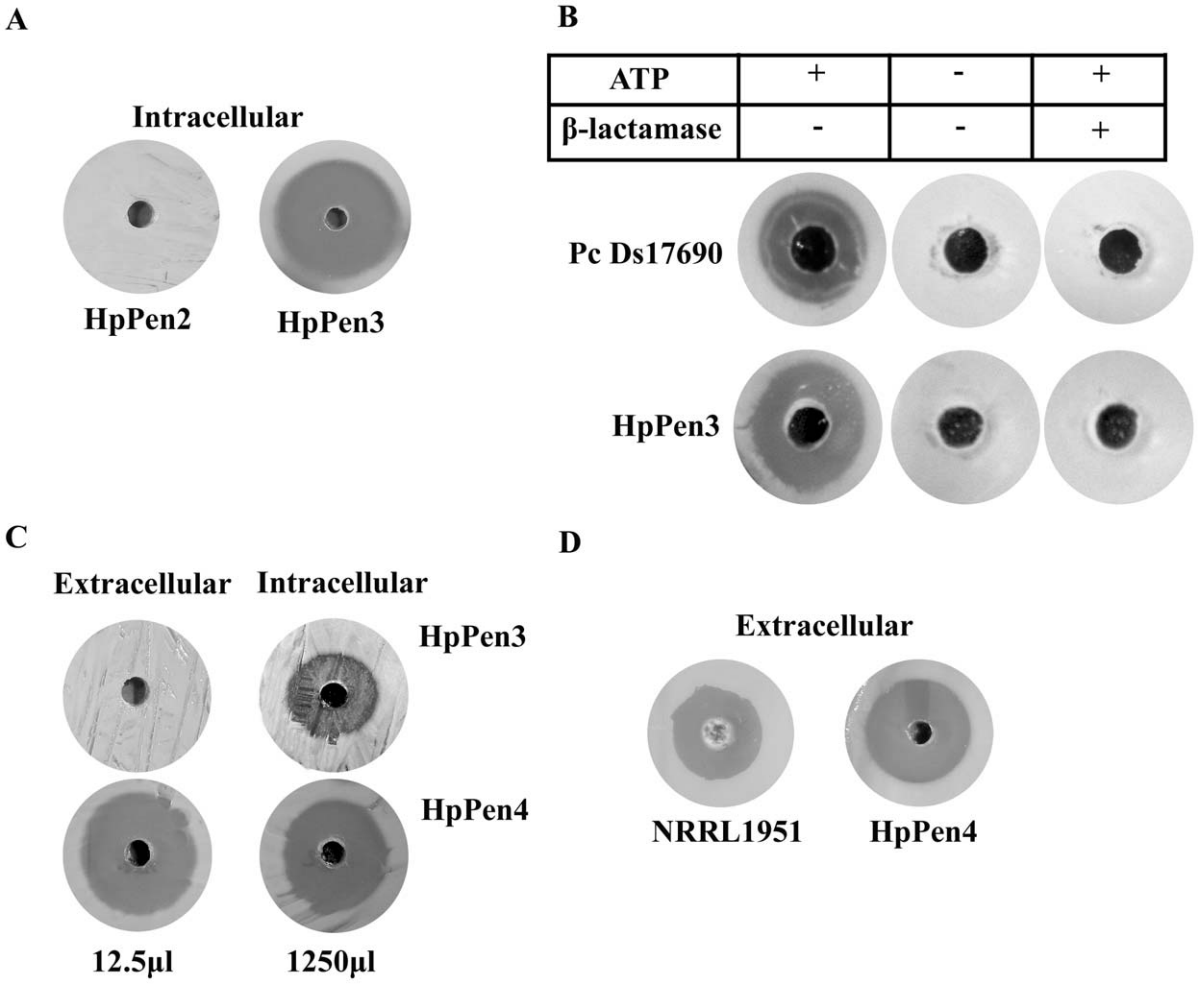
Production of β-lactam antibiotics in *H. polymorpha.* (A) *In vivo* production of IPN by HpPen3 cells. HpPen3 cells were grown in a methanol-limited chemostat in the presence of 8 mM AAA. Cell extracts (corresponding to 1250 µl culture) were loaded in a well of a bioassay plate, which had been overlayed with the indicator strain *M. luteus*. After incubation of the plate, a halo, representing a zone of growth inhibition, was observed indicating that an antibiotic compound was produced. A halo was not observed when an extract of identically grown cells of the HpPen2 control strain were used. (B) *In vitro* production of IPN using HpPen3 cell extracts. Cell extracts were prepared from methanol grown HpPen3 cells and a volume corresponding to 500 microgram of protein was used for *in vitro* IPN synthesis. As a control, a desalted cell extract of *P. chrysogenum* DS17690 cells (250 µg protein) was used. Extracts were incubated in the presence of ATP and the amino acids AAA, L-cysteine, L-valine and subsequently loaded in a well of a bioassay plate, which had been overlayed with the indicator strain *M. luteus*. After incubation of the plate, a halo was observed indicating that an antibiotic compound was produced. This halo was absent in the control experiments performed without ATP or when β-lactamase was added to the reaction mixture. (C) Secretion of antibiotic compounds by HpPen4 cells. HpPen3 and HpPen4 cells were grown in continuous cultures on a mixture of glucose and methanol in the presence of 1 mM AAA and 1 mM PAA. A small aliquot (12.5 µl) of the spent medium of HpPen4, but not HpPen3 cultures, resulted in growth inhibition of the indicator strain (panels marked extracellular) on bioassay plates. Using crude cell extracts (panels marked intracellular) of HpPen3 and HpPen4 cells antibiotic compounds were detected as well. The amount of crude extracts used for the bioassay corresponded to 1250 µl of the culture volume. (D) HpPen4 cells secrete comparable amounts of antibiotics relative to *P. chrysogenum* NRRL1951. HpPen4 cells were grown in batch cultures on methanol in the presence of 1 mM PAA and 1 mM AAA. *P. chrysogenum* NRRL1951 cells were grown in batch cultures on production medium in the presence of 3 mM PAA. The figure shows that similar amounts of antibiotics are secreted by both organisms. 25 µl spent medium of both cultures was used.

Subsequently, we introduced *P. chrysogenum* IPNS in HpPen2 to produce IPN, the next intermediate of the PEN biosynthesis pathway [6] (Fig. 1). In the resulting strain (HpPen3), ACVS, Sfp and IPNS were properly produced (Fig. 3). Upon growth of these cells in medium containing AAA, intracellular accumulation of a β-lactam antibiotic (presumably IPN) could be demonstrated in a bioassay using the β-lactam sensitive indicator strain *Micrococcus luteus* [7] (Fig. 5A). Growth of this indicator strain was not inhibited when an extract was used of similarly grown HpPen2 control cells (Fig. 5A). The presence of enzymatically active ACVS and IPNS in cells of strain HpPen3 to form IPN was confirmed using an *in vitro* assay (Fig. 5B).

### PEN Production in Yeast

As a final step to produce PEN in *H. polymorpha*, we introduced the genes that encode the *P. chrysogenum* peroxisomal enzymes IAT and PCL in HpPen3, resulting in strain HpPen4 (Fig. 3). We previously showed that both heterologous proteins are properly synthesized and sorted in *H. polymorpha* [8], [9]. To test whether HpPen4 cells produced and secreted the β-lactam antibiotic penicillin G (PenG), cells were grown in media supplemented with AAA and phenylacetic acid (PAA), the PenG side chain precursor. As shown in Fig. 5C (extracellular), a clear zone of growth inhibition of *M. luteus* was observed in the bioassay using spent medium of the HpPen4 culture. Growth inhibition was not observed when medium of a control HpPen3 culture was used. This data suggests that PenG is produced and secreted by HpPen4 cells.

**Figure 6:**
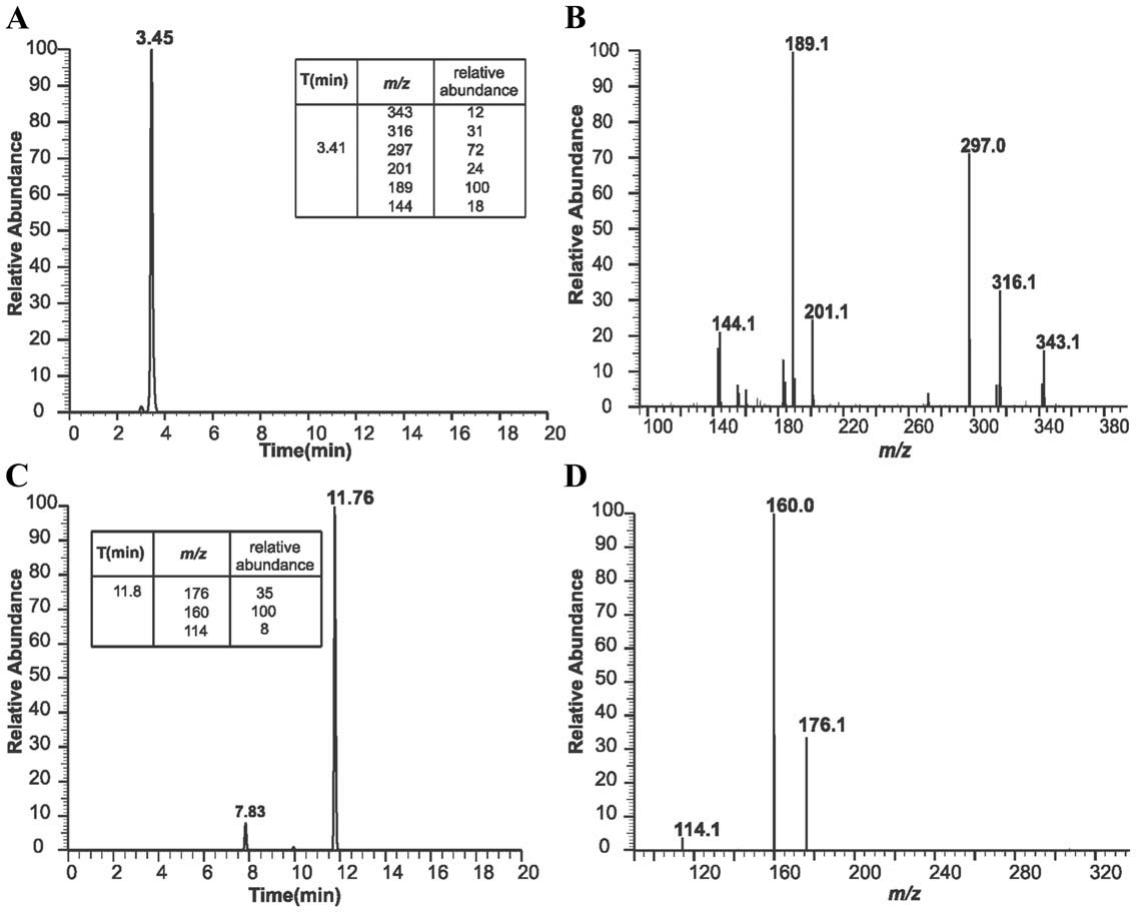
*H. polymorpha* cells produce and secrete PenG: LC MS/MS demonstration of PEN production. Strain HpPen4 was grown in a glucose/methanol-limited continuous culture in the presence of 1 mM AAA and 1 mM PAA. Spent medium was analyzed by LC-MS/MS. The LC chromatograms show the presence of IPN (3.45 min; Fig. 6A) and PenG (11.76 min) and a PenG-related product (7.83 min) (Fig. 6C). Fig. 6B and D show the LC-MS/MS fragmentation patterns corresponding to IPN (Fig. 6B) and PenG (Fig. 6D). The characteristic MS/MS fragmentation pattern of pure IPN and PenG and their relative abundance are represented in the tables present in Fig 6A and C.

LC-MS/MS analyses of spent medium of the HpPen4 culture confirmed the presence of compounds that have the same accurate masses as PenG and IPN (Fig. 6). Furthermore, MS/MS fragmentation patterns of these substances were identical to those obtained using pure IPN or PenG (Fig. 6).

Interestingly, using the same volume of spent media of cultures of *P. chrysogenum* NRRL1951 and HpPen4 halo's of similar size were obtained in the bioassay (Fig. 5C). *P. chrysogenum* NRRL1951 is the strain from which all subsequent high producing strains are derived.

Halo's were also observed when crude extracts were used of HpPen3 or HpPen4 cells, which had been grown in the presence of AAA and PAA (Fig. 5C intracellular). However, extracts corresponding to large culture volumes (100 times more than of the spent medium) had to be used to obtain these halo's (Fig. 5C). In HpPen3 cell extracts the halo is most likely due to the accumulation of IPN, whereas for HpPen4 cells residual amounts of PenG and IPN may remain inside the cells.

### PEN Is Efficiently Secreted by HpPen4 Cells

**Figure 7:**
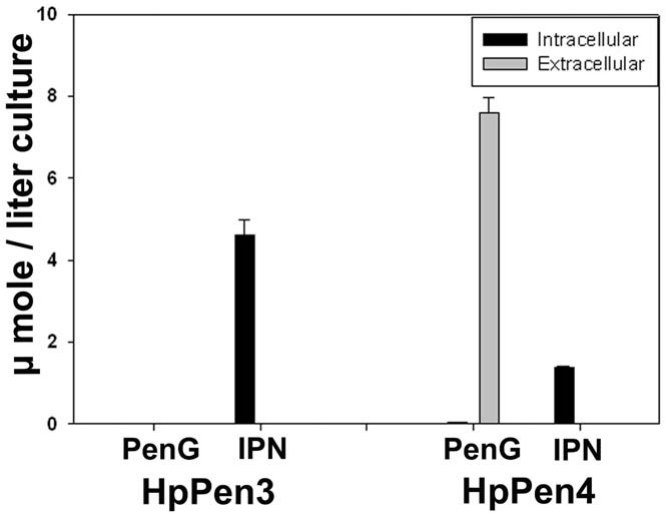
Quantification of produced β-lactam compounds in HpPen3 and HpPen4 cultures. Strains HpPen3 and HpPen4 were grown in glucose/methanol-limited chemostat cultures in the presence of 1 mM AAA and 1 mM PAA. β-lactam compounds were quantified by IP-LC–ESI-ID-MS/MS in spent medium and cell extracts. PenG could not be detected in HpPen3 cultures, but was clearly detectable in HpPen4 cultures, where it was efficiently excreted into the medium. The PenG precursor IPN was present in both HpPen3 and HpPen4 cells; however, the amount was significantly lower in the PenG producing HpPen4 cells. Concentrations are expressed as µmol/l culture. Samples were taken in triplicate. The bars represent the standard error (SE).

Subsequent detailed quantitative analysis of IPN and PenG using ion-pair reversed-phase liquid chromatography–electrospray ionization isotope dilution tandem mass spectrometry (IP-LC–ESI-ID-MS/MS) confirmed that IPN indeed accumulates inside HpPen3 cells (Fig. 7). In identically grown HpPen4 cultures the level of intracellular IPN was reduced relative to those in HpPen3 cells. However, these cells produced PenG, which was predominantly present extracellularly, confirming that PenG is secreted by HpPen4 cells. Calculation of the intracellular metabolite concentrations revealed that PenG is efficiently secreted by HpPen4 cells (the ratio of the extracellular/intracellular concentration of PenG is 24). However, how PenG and its intermediates are transported over the peroxisome and plasma membrane is still largely speculative. Recently, the presence of a regulatable porin in the peroxisomal membrane has been described [10] that could be involved in this process. The HpPen4 cells also produced low concentrations of 6-amino penicillinic acid (6-APA), which was barely detectable in HpPen3. In HpPen4 cells 6-APA is most likely formed by hydrolysis of IPN [11]. In line with the analyses shown in Fig. 6, IPN was also detectable in the medium of HpPen4 cultures. The amount however was too low to allow accurate quantification using the IP-LC–ESI-ID-MS/MS method used.

### Peroxisomes Are Important for Efficient PEN Production

To analyse the importance of the peroxisome compartment in PEN-producing *H. polymorpha* cells, we analyzed PenG production in HpPen4 cells in which the *PEX3* gene was deleted (Δ*pex3* HpPen4).

**Figure 8:**
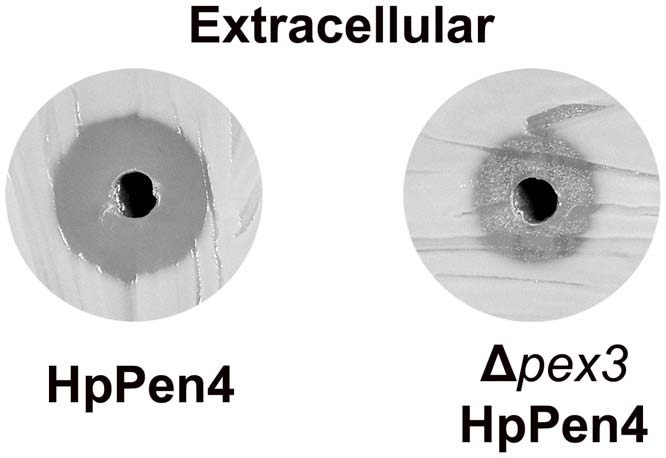
Deletion of *PEX3* results in reduced PEN secretion. HpPen4 and Δ*pex3* HpPen4 cells were grown in a glucose-limited chemostat culture supplemented with choline as nitrogen source in the presence of 1 mM PAA and 1 mM AAA. Using spent medium of Δ*pex3* HpPen4 cultures a smaller halo was produced relative to HpPen4. 6 µl of spent medium of both cultures was used.

Cells were grown on glucose/choline to allow P*_AOX_* induction, as Δ*pex3* HpPen4 cells can not grow on methanol. Deletion of *PEX3* in *H. polymorpha* results in the complete absence of recognizable peroxisomal structures and the mislocalization of all peroxisomal enzymes to the cytosol [12]. Bioassays revealed that a significantly smaller halo was formed using medium of the Δ*pex3* HpPen4 culture relative to HpPen4 medium (Fig. 8). This was confirmed by MS/MS data, which indicated that in glucose/choline grown cells the PenG production had decreased over 50% (1.1 µg/ml in HpPen4 vs 0.4 µg/ml in Δ*pex3* HpPen4). Hence, compartmentalization of IAT and PCL in peroxisomes is important for efficient PenG production in *H. polymorpha*.

## Discussion

Here we present a proof of principle that cells of the yeast *H. polymorpha* can be engineered to produce the important β-lactam antibiotic PEN by introduction of four *P. chrysogenum* genes and one *B. subtilis* gene. This achievement includes several major breakthroughs. First, we show the functional expression of an NRPS in yeast. Secondly, we successfully reconstituted a complex, compartmentalized biosynthetic pathway in yeast that is catalyzed by cytosolic and peroxisomal enzymes and requires transport of precursors, intermediates and end products across the peroxisomal and plasma membrane (Fig. 1). Finally, we demonstrate that intact peroxisomes are required for efficient product formation.

Functional expression of the NRPS ACVS in *H. polymorpha* was dependent on the function of a heterologous PPTase, to modify the protein by covalent binding of phosphopantetheinyl moieties. Apparently, the endogenous *H. polymorpha* PPTase (Lys5) is unable to activate ACVS, whereas the broad specificity bacterial enzyme is suitable for this modification and functional *in vivo* in *H. polymorpha*. While this paper was under review [13] published a similar result, expressing functional ACVS in baker's yeast.

Functionally expression of an NRPS in yeast opens the way to explore novel and improved processes to produce other NRPS-based natural peptides in this cell factory. NRPS's allow synthesis of a broad range of valuable compounds, including almost all peptide-based antibiotics as well as other clinically important compounds such as immunosuppressors and anti-tumor compounds [14].

Our data open the way to use yeast as a host organism for NRPS engineering. NRPS engineering is a strongly emerging field [15], [16] with ample opportunities to develop novel products and processes. These developments rely on efficient NRPS production systems that have now become available in yeast. A single NRPS consist of an arrangement of modules, in which each module is responsible for the incorporation and modification of one amino acid building blocks in the growing polypeptide chain. This modular structure of NRPS's renders them highly attractive targets for protein engineering by domain swapping or active-site modifications. Taking advantage of the power of yeast genetics and handling, our finding offers important novel options that highly facilitate NRPS engineering.

In *P. chrysogenum*, PEN production is compartmentalized in peroxisomes and the cytosol. The efficient secretion of PEN produced in *H. polymorpha* indicates that highly dedicated *P. chrysogenum* transporter proteins apparently are not required for the intracellular transport processes, for uptake of PAA and AAA, nor for the efficient secretion of the end product. Also, compartmentalization is important for efficient PEN production, as the PEN production levels were reduced in a peroxisome deficient (Δ*pex3*) background. In *Aspergillus nidulans* a relation between peroxisome function and PEN production was observed as well [17]. Why the peroxisomal localization of IAT and PCL is important, remains however obscure.

The current non-optimized *H. polymorpha* strain produces similar amounts of PEN as the original *P. chrysogenum* strain (NRRL1951). Future up-scaling programs will strongly benefit from the advanced molecular toolbox available for *H. polymorpha* [2], [18], [19] that is not yet available for *P. chrysogenum*. These approaches can also be used to rapidly develop novel antibiotics by introducing additional heterologous genes and high throughput screening procedures. The development of novel antibiotics is extremely important because of the continuous combat against antibiotic resistant bacteria. Because *H. polymorpha* grows well on various cheap carbon sources (e.g. methanol), the newly engineered cell factory also enables producing these components from alternative and sustainable feedstocks.

## Materials and Methods

### Microorganisms and Growth

The *H. polymorpha* strains used are derivatives of NCYC495 *ade11.1 leu1.1* [20] and listed in Table 1. All plasmids are indicated in Table 2.

**Table 1:** *H. polymorpha* strains used in this study.

Strain	Description	Reference
WT	NCYC495 *ade11.1 leu1.1 ura3 met6*	[8]
HpPen1	WT with integrated P*_AOX_*-*pcbAB*-T*_AMO_* cassette	This study
HpPen2	HpPen1 with integrated P*_TEF1_*-*sfp.His6*-T*_AMO_* cassette	This study
HpIPNS #4.2	NCYC495 *ade11.1 leu1.1* with integrated P*_AOX_*-*pcbC*-T*_AMO_* cassette	[6]
HpIPNS.ACVS	HpIPNS #4.2 with integrated P*_AOX_*-*pcbAB*-T*_AMO_* cassette	This study
HpPen3	HpIPNS.ACVS with integrated P*_TEF1_*-*sfp.His6*-T*_AMO_* cassette	This study
HpPen4	HpPen3 with integrated P*_AOX_*-*penDE*-T*_AMO_* and P*_AOX_-pclA^SKL^*-T*_AMO_* cassettes	This study
Δ*pex3*.HpPen4	HpPen4 with deletion of *PEX3* gene	This study

**Table 2:** Plasmids used in this study.

Plasmid	Description	Source/reference
pHIPG4U	Plasmid containing *H. polymorpha AOX* promoter and *AMO* terminator regions; *H. polymorpha URA3* and G-418^R^ markers; Kan^R^	Lab collection
pME1213	Plasmid containing *P. chrysogenum pcbAB* gene; Amp^R^	Lab collection
pG4U-pcbAB	pHIPG4U with *P. chrysogenum pcbAB* gene under control of *H. polymorpha AOX* promoter; *H. polymorpha URA3* and G-418^R^ markers; Kan^R^	This study
pHIPZ4	Plasmid containing *H. polymorpha AOX* promoter and *AMO* terminator regions; Zeo^R^ marker; Amp^R^	[33]
pZ4-pcbAB	pHIPZ4 with *P. chrysogenum pcbAB* gene under control of *H. polymorpha AOX* promoter; Zeo^R^; Amp^R^	This study
pQE60-Sfp.His6	*Escherichia coli* plasmid expressing *B. subtilis sfp.His6* gene; Amp^R^	Lab collection
pHIPA4	Plasmid containing *H. polymorpha AOX* promoter and *AMO* terminator regions; *H. polymorpha ADE11*; Amp^R^	[34]
pHIPX7	Plasmid containing *H. polymorpha TEF1* promoter and *AMO* terminator regions; *S. cerevisiae LEU2*; Kan^R^	[35]
pA4-Sfp.His6	pHIPA4 with *B. subtilis sfp.His6* gene under control of *H. polymorpha AOX* promoter; *H. polymorpha ADE11*; Amp^R^	This study
pA7-Sfp.His6	pHIPA7 with *B. subtilis sfp.His6* gene under control of *H. polymorpha TEF1* promoter; *H. polymorpha ADE11*; Amp^R^	This study
pPIC6-A	*Pichia pastoris* integration vector; Bla^R^, Amp^R^	Invitrogen
pHP-penDE	Plasmid containing *P. chrysogenum penDE* gene under control of *H. polymorpha AOX* promoter; *S. cerevisiae LEU2*; Kan^R^	[9]
pSNA04	Plasmid containing *H. polymorpha pex3::nat* deletion cassette; Nat^R^; Amp^R^	[36]
pB4-IAT	Plasmid containing *P. chrysogenum penDE* gene under control of *H. polymorpha AOX* promoter; Bla^R^, Amp^R^	This study
pHIPM4-PCL^SKL^	Plasmid containing *P. chrysogenum pcl^SKL^* gene under control of *H. polymorpha AOX* promoter; *H. polymorpha MET6*; Kan^R^	[8]
pBM4-IAT.PCL^SKL^	pHIPM4-PCL^SKL^ containing *P. chrysogenum penDE* gene under control of *H. polymorpha AOX* promoter; *H. polymorpha MET6*; Bla^R^, Kan^R^	This study

**Key:** Amp^R^, ampicillin resistant; Bla^R^, blasticidine resistant; G-418^R^, geneticin resistance; Kan^R^, kanamycin resistant; Nat^R^, nourseothricin resistant; Zeo^R^, zeocin resistant.

Yeast cells were grown at 25°C in batch cultures on 0.5% methanol for 36 hours [6] or in carbon-limited chemostat cultures [21], [22]. Chemostat cultures were grown at a dilution rate of 0.1 h^−1^, pH 5.0. The feed contained a mixture of glucose (0.25%) and methanol (0.2%) [22] or glucose (0.25%) and choline (0.2%) [21].

*P. chrysogenum* strains DS17690 [23] and NRRL1951 [24] were grown in batch cultures on a defined PEN production medium supplemented with 3 mM PAA [25]. Micrococcus *luteus* ATCC 9341 was used for bioassays and grown on 2 x YT agar plates containing 2% bacto-tryptone, 1% yeast extract and 1% NaCl at 30°C.

### Construction of Plasmids

#### Plasmid pZ4-pcbAB

To facilitate cloning of the *P. chrysogenum pcbAB* gene, a *Hind*III site was introduced upstream of the start codon by PCR with the primers ACVS-forward (5′ AGAAAGCTTATGACTCAACTGAAGCCAAA-3′) and ACVS reverse (5′ CTTATCTAGAAACAATGCGAC 3′) using plasmid pME1213 as template. Subsequently, the resulting 1.1 kb product was cloned as a *Hind*III-*Xba*I fragment into the *Hin*dIII+*Xba*I-digested vector pHIPZ4. The resulting plasmid was digested with *Xba*I+*Sma*I and an *Xba*I-*Sma*I fragment of plasmid pME1213, containing the remainder of the *pcbAB* gene, was inserted. The resulting plasmid, designated pZ4-pcbAB, contains the entire *P. chrysogenum pcbAB* gene flanked by the inducible *H. polymorpha* alcohol oxidase promoter (P*_AOX_*) and the amine oxidase terminator (T*_AMO_*).

#### Plasmid pG4U-pcbAB

A 13,777 bp *Not*I-*Sma*I fragment of plasmid pZ4-pcbAB, containing the *P. chrysogenum pcbAB* gene and the *H. polymorpha* P*_AOX_* region, was inserted into *Sal*I (blunted by Klenow treatment) + *Not*I-digested plasmid pHIPG4U. The resulting plasmid, designated pG4U-pcbAB, contains the entire *P. chrysogenum pcbAB* gene flanked by the *H. polymorpha* P*_AOX_* and T*_AMO_* regulatory sequences, a dominant selection marker conferring resistance to G-418 and the *H. polymorpha URA3* gene for targeted integration.

#### Plasmid pA7-Sfp.His6

We first amplified the *B. subtilis sfp.His6* fusion gene with primers Sfp-F (5′ GCGGATCCATGAAGATTTACGGAATTTATATGG 3′) and Sfp-R (5′ TCGGTC GACTTAGTGATGGTGATGGTGATGAGATC 3′) using plasmid pQE60-Sfp.His6 as template. The resulting PCR product was then inserted as a *BamH*I-*Sal*I fragment into *Bam*HI+*Sal*I-digested plasmid pHIPA4, resulting in plasmid pA4-Sfp.His6. In order to have constitutive expression of the *sfp.His6* gene, the *H. polymorpha* P*_AOX_* region of pA4-Sfp.His6 (flanked by *Not*I and *BamH*I sites) was replaced by a *Not*I-*BamH*I fragment containing the *H. polymorpha TEF1* promoter of pHIPX7. The resulting plasmid, pA7-Sfp.His6, contains the *B. subtilis sfp.His6* gene flanked by the *H. polymorpha* P*_TEF1_* and T*_AMO_* regulatory sequences and the *H. polymorpha ADE11* gene as auxotrophic marker.

#### Plasmid pBM4-IAT.PCL^SKL^

First, we isolated the blasticidine gene from plasmid pPIC6-A as a 1969 bp *BamH*I (blunted by Klenow treatment)-*Bgl*II fragment and cloned that between the *Spe*I (blunted by Klenow treatment) and *Bgl*II sites of plasmid pHP-penDE resulting in plasmid pB4-IAT. In order to clone the *pclA* gene encoding *P. chrysogenum* phenylacetyl CoA ligase in plasmid pB4-IAT, plasmid pHIPM4-PCL^SKL^ was digested with *Spe*1 and the resulting 6146 bp fragment was ligated into *Xba*I-linearized pB4-IAT, resulting in plasmid pBM4-IAT.PCL^SKL^. In this plasmid the *P. chrysogenum penDE* and *pclA^SKL^* genes are both controlled by *H. polymorpha* P*_AOX_* and T*_AMO_* regulatory sequences.

### Construction of *H. polymorpha* Strains

#### HpPen1 and HpPen2

A *H. polymorpha* strain producing ACVS (strain HpPen1) was constructed as follows: Plasmid pG4U-pcbAB was linearized with *Bst*1107I in the *H. polymorpha URA3* region and transformed into *H. polymorpha* NCYC495 *ade11.1 leu1.1 ura3 met6*. Uracil-prototrophic transformants were selected and also tested for G-418 resistance (50 µg/ml). A strain producing ACVS was designated HpPen1.

Strain HpPen2 was generated by integration of *Nde*I-linearized plasmid pA7-Sfp.His6 in the genome of HpPen1 with selection on adenine prototrophy and production of both ACVS and Sfp.His6.

#### HpPen3 and HpPen4

For the purpose of generating an *H. polymorpha* strain expressing all PEN biosynthesis genes, plasmid pZ4-pcbAB was digested with *Ssp*I and used to transform the *H. polymorpha* strain HpIPNS #4.2. Transformants were selected on YPD plates that contained zeocin (300 µg/ml). A strain producing both ACVS and IPNS was designated HpIPNS.ACVS. For construction of HpPen3, plasmid pA7-Sfp.His6 was digested with *Not*I and *Kpn*I and the resulting 4656 bp DNA fragment was used to transform strain HpIPNS.ACVS. Adenine prototrophic transformants were selected. A transformant that produced ACVS, Sfp.His6 and IPNS was designated HpPen3.

For the purpose of stable integration of the *P. chrysogenum pcbDE* and *pclA^SKL^* genes, plasmid pBM4-IAT.PCL^SKL^ was linerarized using *Nde*I in the *MET6* locus. The linerarized plasmid was then transfomed into strain HpPen3. Transformants were selected on YPD plates with blasticidine (300 µg/ml). A strain producing all five PEN enzymes was designated HpPen4.

#### Δpex3.HpPen4

In order to create a peroxisome deficient derivative of HpPen4, the *pex3::nat* deletion cassette was PCR amplified from plasmid pSNA04 with primers pex3-nat-fw ( 5′ ACCGCGTGAAACTTTATATCG 3′) and pex3-nat-rev (5′ CAAGGAACGCGATGTATGTT 3′). The resulting 1923 bp fragment was used to transform HpPen4. Transformants were selected on YPD plates with nourseothricin (100 µg/ml). Correct deletion of *PEX3* was confirmed using Southern blot analysis. The resulting strain was designated Δ*pex3*.HpPen4.

### Biochemical Methods

Crude extracts of *H. polymorpha* cells [26] and *P. chrysogenum* hyphae [27] were prepared as described previously. Protein concentrations were determined using the Bio-Rad Protein Assay system using bovine serum albumin as a standard. Western blots were prepared using extracts of *H. polymorpha* and *P. chrysogenum* cells, obtained using the TCA method [27], and decorated using antibodies raised against IPNS, ACVS, IAT, PCL [8], [28], or the His6 tag (Santa Cruz Biotechnology, INC.).

### Bioassays

The presence of β-lactams was analysed by a bioassay using agar plates on which *M. luteus* cells were plated. Samples were loaded in wells in the plates and growth was monitored upon overnight incubation at 30°C. To detect the formation of β-lactams *in vitro*, crude extracts were prepared in buffer A containing 100 mM Tris-HCl pH 8.0, 20% glycerol, 2 mM DTT, 25 mM KCl and 1 mM PMSF. Small molecules were removed from *P. chrysogenum* crude extracts by gel filtration using a PD-10 column. Extracts were incubated at 25°C in buffer A supplemented with 5 mM AAA, 1 mM L-cysteine, 5 mM L-valine, 5 mM ATP, 20 mM MgCl_2_, 0.43 mM FeSO_4._ 7H_2_O and 14.1 mM L-ascorbic acid. After 60 min of incubation, the reaction was terminated by addition of 5 mM of EDTA pH 8.0. The presence of β-lactams was monitored using the bioassay as detailed above. As a control, samples were incubated with β-lactamase (50,000 IU per reaction) prior to termination of the reaction.

### Detection of Metabolites

10 ml of chemostat broth was sampled directly into a filtration beaker containing 50 ml of a −40°C 60% v/v aqueous methanol quenching solution. The quenched cells were filtered over a glass fiber filter (type A/E, Pall Corporation, East Hills, NY, USA, 47 mm diameter, 1 µm pore size) and was washed with 2×50 ml of the quenching solution to completely remove extracellular metabolites. For accurate quantification purposes by IDMS [29] 100 µl of ^13^C internal standard solution was added to the cells. This ^13^C internal standard solution contained all relevant metabolites as U-^13^C-labeled isotopes. Immediately after the cells were submerged in a tube containing 30 ml of a 73°C 75% v/v aqueous ethanol solution. The tube was vigorously mixed and transferred to a water bath at 95°C for 5 min for extraction of metabolites and inactivation of enzymes. The extract was concentrated under vacuum and analysed via ion-pair reversed-phase liquid chromatography-isotope dilution tandem mass spectrometry (IP-LC-ESI-ID-MS/MS) as described previously [30]. The same analysis method was used to determine extracellular metabolite concentrations in quenched and filtered chemostat broth, which was obtained as described previously [31]. The concentration of intracellular metabolites were calculated, assuming a cellular liquid content of 2.38 ml per g dry weight [32].

### Mass Spectrometry Analysis of β-Lactams

For liquid chromatographical analysis, samples were separated on a C18 capillary column (Waters sunfire C18, 2.1×150 mm, 1.8 µm particle size LC column, Waters Chromatography B.V The Netherlands) coupled to an Accela pump (Thermo Electron Corporation). The injection volume was 25 µl, the flow rate 200 µl/min and the elution temperature 30°C. Gradients were prepared using solution A (20 mM ammonium formate in milli Q water) and solution B (a 1∶1 mixture of 20 mM ammonium formate in milli Q water and acetonitrile). A gradient of 5–50% solution B was ran during 12 min, followed by a gradient of 50–65% solution B during 3 min, a washing step with a gradient of 65–5% solution B during 3 min followed by regeneration of the column with 5% solution B. As controls, standard IPN (synthesized by Syncom B.V., the Netherlands) and PenG (Sigma Aldrich) solutions were analysed to determine their respective retention times. Following LC separation, the eluates were directly analysed by mass determination using an LTQ orbitrap (Thermo Electron Corporation). MS data acquisition was performed in positive ion mode. For structure determination MS/MS was performed using LTQ XL (Thermo Electron Corporation) with an *m/z* range of 200–1000.

## References

[1] Kresse H, Belsey MJ, Rovini H (2007). The antibacterial drugs market.. Nat Rev Drug Discov.

[2] Stockmann C, Scheidle M, Dittrich B, Merckelbach A, Hehmann G (2009). Process development in *Hansenula polymorpha* and *Arxula adeninivorans*, a re-assessment.. Microb Cell Fact.

[3] Evers ME, Trip H, van den Berg MA, Bovenberg RA, Driessen AJ (2004). Compartmentalization and transport in beta-lactam antibiotics biosynthesis.. Adv Biochem Eng Biotechnol.

[4] Walsh CT, Gehring AM, Weinreb PH, Quadri LE, Flugel RS (1997). Post-translational modification of polyketide and nonribosomal peptide synthases.. Curr Opin Chem Biol.

[5] Mootz HD, Schorgendorfer K, Marahiel MA (2002). Functional characterization of 4′-phosphopantetheinyl transferase genes of bacterial and fungal origin by complementation of *Saccharomyces cerevisiae lys5*.. FEMS Microbiol Lett.

[6] Gidijala L, Bovenberg RA, Klaassen P, van der Klei IJ, Veenhuis M (2008). Production of functionally active *Penicillium chrysogenum* isopenicillin N synthase in the yeast *Hansenula polymorpha*.. BMC Biotechnol.

[7] Ramos FR, Lopez-Nieto MJ, Martin JF (1985). Isopenicillin N synthetase of *Penicillium chrysogenum*, an enzyme that converts delta-(L-alpha-aminoadipyl)-L-cysteinyl-D-valine to isopenicillin N.. Antimicrob Agents Chemother.

[8] Gidijala L, van der Klei IJ, Veenhuis M, Kiel JA (2007). Reprogramming *Hansenula polymorpha* for penicillin production: expression of the *Penicillium chrysogenum pcl* gene.. FEMS Yeast Res.

[9] Lutz MV, Bovenberg RA, van der Klei IJ, Veenhuis M (2005). Synthesis of *Penicillium chrysogenum* acetyl-CoA:isopenicillin N acyltransferase in *Hansenula polymorpha*: first step towards the introduction of a new metabolic pathway.. FEMS Yeast Res.

[10] Rokka A, Antonenkov VD, Soininen R, Immonen HL, Pirila PL (2009). Pxmp2 is a channel-forming protein in Mammalian peroxisomal membrane.. PLoS One.

[11] Demain A (1983). Biosynthesis of β-lactam antibiotics..

[12] Baerends RJ, Rasmussen SW, Hilbrands RE, van der Heide M, Faber KN (1996). The *Hansenula polymorpha PER9* gene encodes a peroxisomal membrane protein essential for peroxisome assembly and integrity.. J Biol Chem.

[13] Siewers V, Chen X, Huang L, Zhang J, Nielsen J (2009). Heterologous production of non-ribosomal peptide LLD-ACV in *Saccharomyces cerevisiae*.. Metab Eng.

[14] Konz D, Marahiel MA (1999). How do peptide synthetases generate structural diversity?. Chem Biol.

[15] Mootz HD, Schwarzer D, Marahiel MA (2002). Ways of assembling complex natural products on modular nonribosomal peptide synthetases.. Chembiochem.

[16] Cane DE, Walsh CT, Khosla C (1998). Harnessing the biosynthetic code: combinations, permutations, and mutations.. Science.

[17] Sprote P, Brakhage AA, Hynes MJ (2009). Contribution of peroxisomes to penicillin biosynthesis in *Aspergillus nidulans*.. Eukaryot Cell.

[18] van Dijk R, Faber KN, Kiel JA, Veenhuis M, van der Klei I (2000). The methylotrophic yeast *Hansenula polymorpha*: a versatile cell factory.. Enzyme Microb Technol.

[19] Gellissen G, Kunze G, Gaillardin C, Cregg JM, Berardi E (2005). New yeast expression platforms based on methylotrophic *Hansenula polymorpha* and *Pichia pastoris* and on dimorphic *Arxula adeninivorans* and *Yarrowia lipolytica* - a comparison.. FEMS Yeast Res.

[20] Gleeson MAG, Sudbery PE (1988). Genetic analysis in the methylotrophic yeast *Hansenula polymorpha*.. Yeast.

[21] Zwart KB, Veenhuis M, Harder W (1983). Significance of yeast peroxisomes in the metabolism of choline and ethanolamine.. Antonie Van Leeuwenhoek.

[22] van der Klei IJ, Harder W, Veenhuis M (1991). Methanol metabolism in a peroxisome-deficient mutant of *Hansenula polymorpha*: a physiological study.. Arch Microbiol.

[23] Kleijn RJ, Liu F, van Winden WA, van Gulik WM, Ras C (2007). Cytosolic NADPH metabolism in penicillin-G producing and non-producing chemostat cultures of *Penicillium chrysogenum.*. Metab Eng.

[24] Waksman SA, Reilly HC (1944). Strain Specificity and Production of Antibiotic Substances: III. *Penicillium Notatum-Chrysogenum* Group.. Proc Natl Acad Sci U S A.

[25] Hillenga DJ, Versantvoort HJ, Driessen AJ, Konings WN (1994). Structural and functional properties of plasma membranes from the filamentous fungus Penicillium chrysogenum.. Eur J Biochem.

[26] Waterham HR, Titorenko VI, Haima P, Cregg JM, Harder W (1994). The *Hansenula polymorpha PER1* gene is essential for peroxisome biogenesis and encodes a peroxisomal matrix protein with both carboxy- and amino-terminal targeting signals.. J Cell Biol.

[27] Kiel JA, van der Klei IJ, van den Berg MA, Bovenberg RA, Veenhuis M (2005). Overproduction of a single protein, Pc-Pex11p, results in 2-fold enhanced penicillin production by *Penicillium chrysogenum*.. Fungal Genet Biol.

[28] van der Lende TR, van de Kamp M, Berg M, Sjollema K, Bovenberg RA (2002). delta-(L-alpha-Aminoadipyl)-L-cysteinyl-D-valine synthetase, that mediates the first committed step in penicillin biosynthesis, is a cytosolic enzyme.. Fungal Genet Biol.

[29] Wu L, Mashego MR, van Dam JC, Proell AM, Vinke JL (2005). Quantitative analysis of the microbial metabolome by isotope dilution mass spectrometry using uniformly 13C-labeled cell extracts as internal standards.. Anal Biochem.

[30] Seifar RM, Zhao Z, van Dam J, van Winden W, van Gulik W (2008). Quantitative analysis of metabolites in complex biological samples using ion-pair reversed-phase liquid chromatography-isotope dilution tandem mass spectrometry.. J Chromatogr A.

[31] Mashego MR, van Gulik WM, Vinke JL, Heijnen JJ (2003). Critical evaluation of sampling techniques for residual glucose determination in carbon-limited chemostat culture of *Saccharomyces cerevisiae*.. Biotechnol Bioeng.

[32] Ditzelmuller G, Wohrer W, Kubicek CP, Rohr M (1983). Nucleotide pools of growing, synchronized and stressed cultures of *Saccharomyces cerevisiae*.. Arch Microbiol.

[33] Salomons FA, Kiel JA, Faber KN, Veenhuis M, van der Klei IJ (2000). Overproduction of Pex5p stimulates import of alcohol oxidase and dihydroxyacetone synthase in a *Hansenula polymorpha Pex14* null mutant.. J Biol Chem.

[34] Haan GJ, van Dijk R, Kiel JA, Veenhuis M (2002). Characterization of the *Hansenula polymorpha PUR7* gene and its use as selectable marker for targeted chromosomal integration.. FEMS Yeast Res.

[35] Baerends RJ, Salomons FA, Faber KN, Kiel JA, Van der Klei IJ (1997). Deviant Pex3p levels affect normal peroxisome formation in *Hansenula polymorpha*: high steady-state levels of the protein fully abolish matrix protein import.. Yeast.

[36] Nagotu S, Saraya R, Otzen M, Veenhuis M, van der Klei IJ (2008). Peroxisome proliferation in *Hansenula polymorpha* requires Dnm1p which mediates fission but not de novo formation.. Biochim Biophys Acta.

